# Strategies to Overcome the Barrier of Ischemic Microenvironment in Cell Therapy of Cardiovascular Disease

**DOI:** 10.3390/ijms22052312

**Published:** 2021-02-25

**Authors:** Rouven Berndt, Martin Albrecht, René Rusch

**Affiliations:** 1 Clinic of Cardiovascular Surgery, University Hospital Schleswig-Holstein, 24105 Kiel, Germany; rene.rusch@uksh.de; 2 Vascular Research Center, University Hospital Schleswig-Holstein, 24105 Kiel, Germany; 3 Department of Anesthesiology and Intensive Care Medicine, University Hospital Schleswig-Holstein, 24105 Kiel, Germany; martin.albrecht@uksh.de

**Keywords:** cell therapy, Advanced Therapy Medicinal Product (ATMP), cardiovascular disease, Peripheral Arterial Disease (PAD), Myocardial Infarction (MI), Ischemia/Reperfusion (I/R)

## Abstract

The transplantation of various immune cell types are promising approaches for the treatment of ischemic cardiovascular disease including myocardial infarction (MI) and peripheral arterial disease (PAD). Major limitation of these so-called *Advanced Therapy Medicinal Products (ATMPs)* is the ischemic microenvironment affecting cell homeostasis and limiting the demanded effect of the transplanted cell products. Accordingly, different clinical and experimental strategies have been evolved to overcome these obstacles. Here, we give a short review of the different experimental and clinical strategies to solve these issues due to ischemic cardiovascular disease.

## 1. Introduction

The recent development of gene and cell therapies in cardiovascular disease has given rise to the expectation that the disastrous consequences of occluded vessels in myocardial infarction (MI) and peripheral arterial disease (PAD) could be treated not only by surgical or interventional revascularization, but also by induction of regeneration and angiogenesis in ischemic tissues. However, the transplantation of cells in an ischemic microenvironment means that a number of obstacles to effective treatment have to be overcome [1,2].

Restriction of blood flow due to arterial stenosis/occlusions leads to reduced perfusion of the heart and/or peripheral limbs. Subsequently, an undersupply with oxygen, nutrients, and metabolic substances develops in the area of the sub- or totally occluded arteries with simultaneous accumulation of toxic cell metabolic products [3,4]. To compensate reduced perfusion, angiogenesis is induced in the affected tissues, however, as ischemia progresses, this compensatory capacity is exceeded and tissue ischemia develops [5]. Accordingly, cell death and apoptosis occur in the supply area of the occluded vessel, which, if left untreated, leads to the development of infarction/necrosis zones after some time and chronic ischemic disease [3,4,5]. This process of tissue damage is characterized at the cellular level by damage to the cell membrane, swelling of the mitochondria, and finally rupture of the sarcomeres. In addition, tissue damage is intensified by the increased release of reactive oxygen species (ROS) by leukocytes migrating into the ischemic areas [4,5,6]. Due to the cell damage, local inflammation is induced and circulating monocytes migrate from the bloodstream along a chemotactic gradient into the tissue [7,8]. During this process, a large proportion of monocytes differentiate into M2 macrophages, which predominantly contribute to the debridement of necrotic cells [7,8]. In the course of chronic tissue damage and the resulting inflammatory response, the endothelial barrier function of vessels is also disrupted [4,5,6].

Therefore, any kind of cellular agent for cardiovascular therapy is exposed to oxygen radicals, limited supply of nutrients, and immunological consequences of acute and chronic tissue inflammation. As a consequence, cell viability, therapeutic effect, and retention of the cell product into the ischemic tissues are impaired [9]. Here, we describe several exemplary strategies to improve cell therapy in ischemic tissues with a special focus on cardiovascular disease.

Our search strategy included MEDLINE, EMBASE, and PubMed and a complete list of search terms is given in the annex. In brief, we used a combination of terms that refer to cell therapy in MI or PAD and supporting approaches (e.g., “cardiovascular disease” and “cell delivery device”). To be included in this review, studies had to report on primary research, (meta)data analysis, or recent experiences relating to the search terms, be published in peer-reviewed journals, and be written in English, French, Italian, Spanish or German (as these are the languages spoken by the current authors). The initial search yielded 481 papers, of which 86 contained relevant data and were included in to this review. Table 1 provides an overview of the different therapeutic strategies.

## 2. Cell Priming of Cell Products Prior to Transplantation

### 2.1. Cell Priming by Pro-Angiogenic Factors

**Figure 1:**
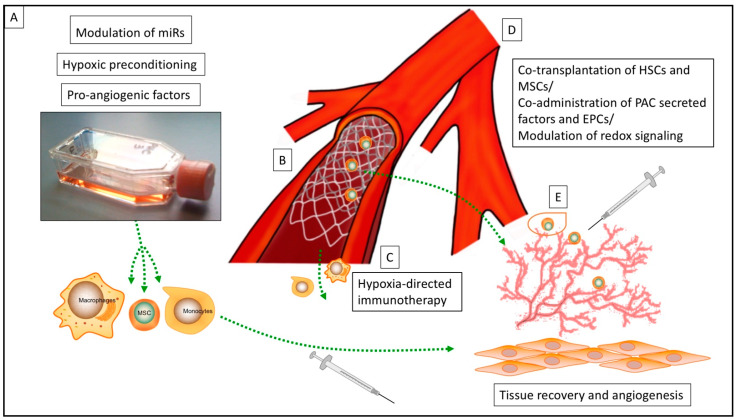
Overview of various strategies for the enhancement of cell therapy for ischemic cardiovascular disease: (**A**) Strategies for cell priming prior to transplantation, (**B**) Cell delivery device (CDD) for intraarterial delivery of the cell products, (**C**) hypoxia-directed immunotherapy, (**D**) strategies for co-transplantation of cell lines and modulation of redox signaling in the ischemic microenvironment, (**E**) encapsulation of cell products prior to transplantation (miR = microRNA; MSC = mesenchymal stem cell; HSC = hematopoietic stem cell; PAC = circulating proangiogenic cell; EPC = endothelial progenitor cell).

A common strategy to enhance cell product efficacy in ischemic cardiovascular disease is the development of preconditioning protocols prior to cell transplantation. The aim of this approach is basically to first improve the viability of the cell product, and second, to enhance the demanded therapeutic effects (Figure 1A). Most experimental strategies focus on increasing the release of pro-angiogenetic proteins by the cell product, predominantly vascular endothelial growth factor (VEGF) via cell programming and culturing methods. Lee and colleagues (2013) demonstrated that a cocktail containing β-mercaptoethanol, all-trans-retinoic acid, basic fibroblast growth factor (bFGF), human platelet-derived growth factor (PDGF)-AA, and heregulin-β1 have the potential to improve VEGF release from human mesenchymal stem cells (MSCs) [10]. A further strategy was introduced by treating endothelial cells and circulating proangiogenic cells (PACs) with a cocktail of pro-angiogenic cytokines including VEGF, stromal cell-derived factor 1 (SDF-1α), and interleukin 8 (IL-8), leading to an increase of nuclear factor E2-related factor 2 (Nrf2). The authors reported that lack of Nrf2 attenuated survival, proliferation, migration, and pro-angiogenic potential of murine PACs and affected the angiogenic transcriptome in vitro. The here described involvement of Nrf2 in neoangiogenesis and its cytoprotective effects revealed a new direction in research on therapeutic neovascularization in cardiovascular disease [11,12].

Immune cells, especially from mononuclear origin, also provide an interesting phenotype and outstanding properties for immune cell transplantation in ischemic disease [13,14]. It is a well-known fact that monocyte migration and macrophages from the reparative type significantly contribute to tissue recovery and angiogenesis in MI and PAD [15,16]. However, the clinical transfer of this knowledge means that a particular cell type has to be generated prior to transplantation. Therefore, so-called programmable cells of monocytic origin (PCMO) and regulatory macrophages (Mreg) have been described as promising cell types for transplantation into ischemic tissues [17]. Both cell types can be generated from leukapheresis products and cultured similarly with macrophage colony-stimulating factor (M-CSF) and interleukin 3 (Il-3), respectively, with interferon (IFN) γ [18,19]. PCMO and Mreg were designed to overcome the obstacles of ischemic microenvironment showing a robust phenotype in ischemia/reperfusion in vitro experiments and enhanced pro-angiogenic potential by paracrine secretion of macrophage inflammatory protein α (MIP-1 α), granulocyte-macrophage colony-stimulating factor (GM-CSF), pentraxin-related protein 3 (PTX 3), and monocyte chemoattractant protein-1 (MCP-1) in vitro and in vivo studies [17,18,19]. Moreover, cell lines from monocytic origin may provide further cellular features that could also support or induce tissue regeneration due to the reparative capabilities and phagocytic activity of mononuclear cells. Transplantation of PCMO into chronic ischemic heart and hind limb in mice contributed significantly to muscle recovery most likely mediated by paracrine secretion of GM-CSF [17,18]. This might be a relevant aspect in the development of cell products from mononuclear cells and in the treatment of chronic ischemic cardiovascular disease, especially in patients with PAD IV-V (Rutherford Classification) or “no option” patients with coronary artery disease and MI.

### 2.2. Cell Priming by Modulation of microRNA (miR)

Moreover, top-down and bottom-up experimental strategies have revealed a wide range of molecular targets for the enhancement of therapeutic cell properties in ischemic microenvironment. Recently, the modulation of several microRNAs (miRs) in pro-angiogenic cell lines has been reported as a sufficient strategy for the enhancement of angiogenic properties and survival rates of transplanted cells (Figure 1A). Besnier et al. (2018) reported that the increase of miR-210 by hypoxia leads to the repression of Ephrin A3 inducing proangiogenic responses in PACs [20]. Hence, the ex-vivo pre-miR-210 transfection of PACs induced post-ischemic therapeutic neovascularization and blood flow recovery in a mouse limb ischemia model and therefore modulates PAC function and improves their therapeutic potential in PAD. A further study has analyzed twenty-eight miRs potentially able to modulate angiogenesis in patients with PAD; miR-15a and miR-16 were identified as promising therapeutic targets and the improvement of pro-angiogenic cell products. In further studies, transplantation of healthy PACs ex vivo–engineered with anti–miR-15a/16 improved postischemic blood flow recovery and muscular arteriole density in immunodeficient mice. Unfortunately, only a short timeframe of two weeks after initial ischemic event was observed in the in vivo experiments. In line with other experimental approaches, a clear distinction between the therapeutic effect on acute and chronic ischemic tissue damage is not possible and further research appears necessary [21].

### 2.3. Cell Priming by Hypoxia

More recently, hypoxia-based strategies for preconditioning cell lines, mostly MSCs, have also been explored to improve bioactivity and survival under ischemic conditions (Figure A).

A regulatory role of hypoxia-inducible factor (HIF)-proteins on miR expression under hypoxia, especially on miRNA-214 and 210, was reported to be involved in cell survival and proliferation [22,23]. Thus, Lee et al. (2017) investigated the influence of hypoxia preconditioning and underlying mechanisms on MSCs and ascertained that hypoxia-induced 78-kD glucose-regulated protein (GRP78) promoted the proliferation and migration potential of MSCs through the HIF-1α-GRP78-Akt signal axis [24]. After hypoxic preconditioning, the transplanted MSCs showed suppression of the cell death signal pathway and augmentation of angiogenic cytokine secretion in an ischemic hind limb mouse model. Likewise, a recent study examined the therapeutic effects of the hypoxia-induced secretome of MSCs: the authors described that hypoxic preconditioning induced secretion of MSCs enhanced cell viability and angiogenesis and promoted wound healing in a gastric ulcer model in rats. Activation of the cyclooxygenase (COX)-prostaglandin E (PGE) 2 axis being mediated by the extracellular signal-regulated kinases (ERK) 1/2 pathway was discovered as the underlying mechanism in this study [25]. A large number of similar trials have supported these results, but without making a significant step toward clinical translation and underlining that potentially, the hypoxia-induced cell-free secretome itself might be a more sufficient solution for a potential clinical application [26,27,28].

Equally, the utilization of hypoxic cell priming was introduced to the experimental treatment of acute and chronic ischemic heart disease. Hypoxia-conditioned medium derived from hypoxia treated bone marrow MSCs was used in a heart transplantation model to prevent the ischemia/reperfusion injury and consecutive heart failure after heart transplantation. A cardioprotective effect was observed and attributed to the phosphoinositide 2/3-kinases-Akt (PI2K/PI3K-Akt) signaling pathway, but the authors also mentioned that their study was unable to precisely identify the underlying cytokine signaling responsible for the beneficial effects, emphasizing again the unsolved issues of cell priming [29]. Further studies investigating the cardioprotective effects of secretome from various hypoxia-conditioned cell lines have supported these findings and on putting further emphasis on HIF-1α mediated pathways [30,31,32].

Finally, concerns remain if approaches of hypoxic-preconditioning could be sufficiently transferred into clinical practice. The permanent effect of hypoxia on cell products has not been investigated so far and only data from basic research are actually available. The clinical use of instant hypoxic preconditioning, virtually using a hypoxia chamber for bedside application before cell transplantation, remains doubtful due to logistic concerns. It might be speculated whether co-delivery approaches (e.g., transfection of HIF-proteins or therapeutic strategies like *remote ischemic preconditioning* (RIPC)) could be supporting concepts to adapt cell transplantation and ischemic conditioning in clinical practice [33].

## 3. Encapsulation Techniques for Cell Transplants

The development of encapsulation techniques for target orientated drug delivery to organs and delayed relief of drug agents have also influenced the field of cell and gene therapy during the last two decades [34,35]. However, cell products for the treatment of PAD or critical limb ischemia have demonstrated only modest results or failed in clinical studies due to poor cell retention within the ischemic microenvironment [36,37,38]. During the last decade, a research group from *King’s College London* has continually evolved a *Good Manufacturing Practice* (GMP)-compliant method for encapsulation of pro-angiogenic macrophages respectively monocytes introducing 300 µm alginate capsules (Figure 1E). Analyses revealed that the encapsulated macrophages did not undergo phenotype switch and preserved their pro-angiogenic potential. The results from in vivo experiments of intramuscular injection into ischemic mouse hind limbs demonstrated increased cell retention, improved pro-angiogenic capacity, and restoration of the chronic ischemic muscle. Moreover, this study is one of the few trial designs providing GMP-compliant solutions for cell therapy in an ischemic microenvironment, gaping the bridge from experimental design to potential clinical application [38,39]. Previous studies have already explored the potential of different encapsulation techniques for cell therapy in cardiovascular disease predominantly describing biocompatible alginate microcapsules or gelatin hydrogels. Interestingly, encapsulation techniques enable application, especially in chronic cardiovascular disease due to the delayed release of reparative and pro-angiogenic cells. Despite auspicious results, encapsulation as a strategy for cell therapy has mostly remained a preclinical concept thus far [40,41,42,43,44].

## 4. Strategies for Ischemia-Directed Guidance of Cell Products

Apart from overcoming the ischemic tissue barrier, there are also a modest number of studies trying to profit from ischemic conditions. The theoretical concept of these pioneering experimental designs is based on the hypoxia-directed guidance of cell products (Figure 1 C). Hypoxia as a stimulus for the monocyte/macrophage axis is well described in numerous physiological and pathological processes (e.g., cardiovascular remodeling, stem cell homing, and rheumatoid arthritis) [45,46,47]. Furthermore, hypoxia-directed guidance of biocompatible nanoparticles, mostly for drug delivery in cancer research, has been reported by various research groups [48,49]. Consequently, hypoxia-directed guidance of cell products appears as the next step. A macrophage-mediated delivery of hypoxia-activated prodrug nanoparticles was introduced in cancer related therapy, but might be open for a wide range of different indications, especially in cardiovascular remodeling [47,50]. The here described mechanism of action, so called “trojan horse strategy” in cell therapy, include nanoparticle loading of macrophages and the chemotactic and phagocytic abilities of the monocyte/macrophage axis to penetrate regions of hypoxia for remodeling processes [50]. In this context, the chemokine (C–C motif) ligand 26 (CCL 26) has been investigated for hypoxia-directed migration of mononuclear cells. The administration of recombinant CCL26 abolished the hypoxia-induced directed migration of human monocytes, while the addition of CCL26 under normoxic conditions resulted in a repulsion of monocytes from the source of CCL26. Due to its chemorepulsive nature, these findings might be directly linked with monocyte migration toward hypoxia and might be a promising target for hypoxia-directed immunotherapy, not only for cancer, but also for cardiovascular therapy [19,51].

## 5. Cell Delivery Devices

Another opportunity to reach the therapeutic region of interest (ROI) besides molecular and cellular modulation is the utilization of so-called “cell delivery devices” (CDDs) (Figure 1B). Mainly, these devices are constructed as a catheter-based technique with consecutive cell product-colonized stent implantation or direct cell injection at the therapeutic ROI (e.g., coronary artery in MI or peripheral femoral artery in critical limb ischemia (CLI)) [52,53,54]. First descriptions of CDD for gen- and cell therapy in cardiovascular disease and cancer therapy appeared two decades ago, but more as a future prospect rather than a real therapy option in the near future [55]. Most CDDs provided a catheter-based cell therapy, enabling the delayed release of VEGF in “no-option” chronic arterial occlusions and consequently the induction of angiogenesis at the therapeutic ROI [52,53,54,56]. Nevertheless, the combination of a cell therapy and a mechanical device has raised several issues—most of them unsolved until today. Cell colonization on devices, cell line modification due to artificial surface interaction, storage, immediate availability in clinical routine, and loss of cell viability due to catheter introduction into the vascular system are only some of these obstacles and as a consequence, despite promising results from animal experiments, none of the described CDDs have made it into clinical translation yet [54,56].

## 6. Cell Transplantation and Modulation of Ischemic Microenvironment

### 6.1. Injection and Transplantation Strategies

In the past, several studies have reported the transplantation of pro-angiogenic cell lines (e.g., stem cells, mononuclear cells, and lymphatic cell lines into ischemic cardiac tissue or muscle) [57,58]. In clinical trials treating PAD with stem cell or immune cell transplantation into the lower limb, the muscle injection sites differed highly and ranged between 10–20 different spots [59,60,61]. Mostly, the angiosome model served as a rationale for cell transplantation, defining an angiosome as an area of tissue comprising skin, subcutaneous tissue, fascia, muscle, and bone supplied by a specific artery and drained by a specific vein [62,63,64]. Hence, injection of the cell product is oriented toward this area of vascular supply with the underlying hypothesis that the injected cells unfold their regenerative potential due to the interaction of migrating macrophages respectively circulating monocytes, and vascular endothelial signaling and sprouting [65,66].

Despite this, no approach has yet provided a standard rationale for cell transplantation in cardiovascular disease [59,60,67]. Accordingly, various authors have reported that the identification of the ischemic/normoxic border zone (i/nBZ) in patients with MI and PAD and consecutive controlled injection of the cell product have the potential to improve the efficacy of the applied cell therapy and to reduce cell doses. Based on the hypothesis that cells directly injected into the therapeutic ROI are poorly incorporated by ischemic tissue, several studies could show the advantages of this strategy (identification of the i/nBZ) in clinical and experimental studies. Shin et al. (2016) demonstrated that transplantation of human MSC into mouse ischemic limbs, in consideration of the i/nBZ, significantly enhanced cell engraftment and secretion of paracrine factors, which effectively stimulated vessel sprouting, enhanced blood perfusion in ischemia/reperfusion injury and enabled the application of significantly reduced cell doses [68]. Impact of the injection-site was also investigated in cell therapy for MI. Transplantation of both bone marrow MSCs into the i/nBZ and the central zone of the MI area contributed to the restoration of heart function. MSCs transplanted into the central zone of MI did not have an initial effect on the recovery of the heart function, but the authors hypothesized that these MSCs contribute to reverse remodeling of ventricular dilation [69].

Despite this, in clinical practice, there are barely diagnostic tools available to enable ROI and/or i/nBZ orientated molecular treatments and most of the so far described approaches are limited to experimental studies. The rapid development of cardiovascular imaging combined with the existing and evolving tools of interventional cardiology, cardiovascular surgery, and radiology could be the next important step in reaching a more individualized cell therapy of cardiovascular disease [70,71].

### 6.2. Modulation of the Ischemic Microenvironment

Modulation of the ischemic microenvironment itself is also required to stimulate neovascularization in PAD (Figure 1D). In particular, a physiological level of oxidants is critical for the engraftment of the neo-vessel, whereas the pathological enrichment of oxidants attenuates vascular growth. Glutaredoxin-1 (Glrx) is an enzyme catalyzing the reversal of so-called S-glutathionylation (GSH adducts) representing a radical scavenger [72,73]. Accordingly, loss of Glrx leads to improvement of vascular growth in vivo whereas Glrx overexpression attenuates VEGF signaling in vitro and ischemic vascularization in vivo [72]. Hence, several Glrx targets including HIF-1α may contribute to inhibition or activation of vascularization by reducing or increasing GSH adducts [73,74]. In animal experiments, it could be demonstrated that enrichment of antioxidants may be counter-productive for the treatment of ischemic disease, and highlights Glrx as a potential therapeutic target in molecular medicine to improve ischemic limb vascularization [73,75,76]. A further strategy of influencing the ischemic microenvironment was introduced by Wang et al. (2021) reporting the blocking of monocyte recruitment by anti-C–C chemokine receptor type 2 (CCR 2). The transplantation of MSCs and subsequently treatment with anti-CCR2 showed promising results in the treatment of acute MI [77]. This could be explained by the initial detrimental effect of migrating monocytes/macrophages, and subsequently cytokine release in the early phase of ischemia related tissue damage, which was potentially abolished in this study [77,78]. Accordingly, accurate control of oxidants is required to stimulate vascularization, while targeting and modulation of i/nBZ might be further promising ways of improving the effect and survival of transplanted cells in ischemic disease.

### 6.3. Stabilization of Vascular Growth and Neo-Vessels

A further challenge in the therapy of cardiovascular ischemic disease is that the induction of neoangiogenesis essentially depends on the precise signaling in the ischemic microenvironment regulated by several exactly balanced factors. Otherwise, the novel developing vascular structures remain immature, so-called tumor-like vessels, with aberrant, thin, and inoperative vessel walls [79,80,81]. In particular, different isoforms of VEGF with varying affinities for extracellular matrix and the concentration of VEGF itself combined with the migration of perivascular cells and vascular smooth muscle cells (SMCs) are essential not only for providing a vascular scaffold, but also for enabling paracrine signaling for vessel sprouting and maturation [79,81].

Thus, different approaches have been developed to orchestrate vascular growth in ischemic tissue. It has been shown that co-culturing of endothelial cells (ECs) with MSCs or fibroblasts and consecutive paracrine secretion by both cell types promote stabilization of neo-vessels [82,83,84,85]. Grigorescu et al. (2015) investigated the therapeutic stabilization of induced neo-vessels in ischemic microenvironments employing an ischemic hind limb mouse model and co-administration of PAC-secreted factors at the time of endothelial progenitor cell (EPC) transplantation (Figure 1D). This procedure improved tissue regeneration and vascular repair through the stabilization of newly-derived blood vessels. The responsible factors identified by the authors included von Willebrand factor (vWF), cadherin 5 (CDH5), multiple EGF-like-domains 10 (MEGF10), early growth response protein 1 (EGR-1), fatty acid binding protein 3 (FABP3), and VEGF [9]. In relation to the cardiac ischemic microenvironment, Lemcke et al. (2017) reported the benefits of co-transplantation of MSCs and hematopoietic stem cells (HSCs) in MI (Figure 1D). Cardiomyogenic plasticity of MSCs was mediated via junction-dependent crosstalk between cardiomyocytes and transplanted stem cells whereas HSCs were involved in the regulation of neoangiogenesis. This approach underlines, first, the importance of investigating cell/tissue interaction after transplantation in detail, and second, that often a more sophisticated concept is necessary instead of mono-cell type transplantation [86].

Finally, various hydrogels including pro-angiogenic factors or mechanobiological approaches have been developed to provide microenvironmental control in ischemic tissue contributing to cell transplantation. Nonetheless, most of these translational strategies resemble rather whole tissue transplantation than a cell therapy and the translational transfer remains questionable [79].

## 7. Final Remarks

In conclusion, most of the here described approaches are still a promise to the future rather than an actual therapy option and currently more bench than bedside. Only a very limited number of the here described approaches and techniques have reached the clinical stage or provided a GMP-compatible strategy. Most researchers and therapists conducting gene and cell therapy are still concerned with questions like “who to treat, which disease, and which gene/cell agent”, despite clinical scientists who should already have an eye on the flanking conditions of the here described ATMPs. What we can already learn from the here described approaches is that in this early stage of experimental and clinical development, it might be the surrounding parameters, thus the knowledge of the ischemic microenvironment, that could decide the success or failure in the treatment of cardiovascular disease.

In the future, it appears that there is a significant step toward more sophisticated and holistic forms of cell therapy defining a priori the demanded cell/tissue interaction, cell delivery, cell retention, and delayed release of active ingredients. To ensure this kind of translational development process, the establishment of specialized centers for the development of ATPMs seems to be a mandatory requirement. Evolving cell therapies of cardiovascular disease might be an important part of the development toward an individualized form of medicine.

## Figures and Tables

**Table 1:** Exemplary overview of therapeutic strategies.

Strategy	Cell Subset	Disease/Model	References
Improvement of injection site of transplanted cells	MSCs	Mouse/Rat Cardiac and hind limb ischemia	[68,69]
Cell Priming by Pro-Angiogenic Factors	MSCs, PACs, PCMO, Mreg, Macrophages, Monocytes	Mouse, rat Cardiac and hind limb ischemia (acute and chronic stage)	[10,11,12,17,18,19]
Transfection of pro-angiogenic cell lines and enhancement of microRNA	PACs	Mouse/hind limb ischemia	[20,21]
Co-administration of PAC secreted factors and EPC, HSCs Co-administration with MSCs and HSCs	PACs, EPCs, MSCs, HSCs	Mouse/hind limb ischemia	[9,86]
Modulation of redox signaling via thiol modification Anti-CCR2 and transplantation of MSCs	MSCs	Mouse/cardiovascular hypertrophy and hind limb ischemia	[72,74,76,77]
Biomechanically defined microenvironment	n.a.	Mouse	[79]
Hypoxic-preconditioning	MSCs	Mouse, rat Cardiac and hind limb ischemia (acute and chronic stage)	[24,28,29]
Encapsulation of cell products	Monocytes, macrophages	Mouse/ hind limb ischemia (GMP compatible protocol) (acute and chronic stage)	[38,39,41,42]
Hypoxia-directed immunotherapy	Monocytes, macrophages	Mouse/ tumor model	[50,51]
Cell delivery devices	EC, MSC, SMC	Swine/ Cardiac and hind limb ischemia	[52,53,54]

## Abbreviations

ATMPs	Advanced Therapy Medicinal Products
CCL26	Chemokine (C-C motif) ligand 26
CCR2	C-C Chemokine Receptor Type 2
CDD	Cell Delivery Device
CDH5	Cadherin 5
COX	Cyclooxygenase
EC	Endothelial Cell
EGR-1	Early Growth Response Protein 1
EPC	Endothelial Progenitor Cells
ERK	Extracellular Signal-regulated Kinases
FABP 3	Fatty Acid Binding Protein 3
Glrx	Glutaredoxin-1
GM-CSF	Granulocyte Macrophage-Colony-Stimulating Factor
GMP	Good Manufacturing Practice
GRP78	78-kD Glucose Regulated Protein
GSH adducts	S-Glutathionylation
HIF	Hypoxia Inducible Factor
HSC	Hematopoietic Stem Cells
IL-3	Interleukin-3
IL-8	Interleukin-8
i/nBZ	Ischemic/normoxic Border Zone
IFN γ	Interferon γ
M-CSF	Macrophage-Colony Stimulating Factor
MSC	Mesenchymal Stem Cells
MCP-1	Monocyte Chemoattractant Protein-1
MEGF10	Multiple EGF-like-domains 10
MI	Myocardial infarction
miR	MicroRNA
MIP-1 α	Macrophage Inflammatory Protein-1α
Mreg	Regulatory Macrophage
PAC	Circulating Proangiogenic Cell
PAD	Peripheral Arterial Disease
PCMO	Programmable Cell of Monocytic Origin
PDGF	Platelet Derived Growth Factor
PEG 2	Prostaglandin E 2
PI2/3K-Akt	Phosphoinositide 2/3-kinases-Akt signaling pathway
PI3K	Phosphoinositide 3-kinases
PTX 3	Pentraxin Related Protein 3
RIPC	Remote Ischemic Preconditioning
ROI	Region of Interest
SDF-1α	Stromal Cell-derived Factor-1α
SMC	Smooth Muscle Cells
VEGF	Vascular Endothelial Growth Factor
vWF	Von Willebrand Factor
**Search terms**	
ATMPs	Advanced Therapy Medicinal Products
Cardiovascular disease	
Cell delivery device	
Cell therapy	
Cell transplantation	
Cytotherapy	
Ischemic microenvironment	
Ischemia/reperfusion	
Myocardial infarction (MI)	
Peripheral arterial disease (PAD)	
Stem cell therapy	

## References

[1] Ylä-Herttuala S., Baker A.H. (2017). Cardiovascular Gene Therapy: Past, Present, and Future. Mol. Ther..

[2] Terashvili M., Bosnjak Z.J. (2019). Stem Cell Therapies in Cardiovascular Disease. J. Cardiothorac. Vasc. Anesthesia.

[3] Bing R.J. (2001). Myocardial ischemia and infarction: Growth of ideas. Cardiovasc. Res..

[4] Jude E.B., Eleftheriadou I., Tentolouris N. (2010). Peripheral arterial disease in diabetes-a review. Diabet. Med..

[5] Simon F., Oberhuber A., Floros N., Busch A., Wagenhäuser M.U., Schelzig H., Duran M. (2018). Acute Limb Ischemia—Much More Than Just a Lack of Oxygen. Int. J. Mol. Sci..

[6] Coats P., Wadsworth R. (2005). Marriage of resistance and conduit arteries breeds critical limb ischemia. Am. J. Physiol. Circ. Physiol..

[7] Grimshaw M.J., Balkwill F.R. (2001). Inhibition of monocyte and macrophage chemotaxis by hypoxia and inflamma-tion—A potential mechanism. Eur. J. Immunol..

[8] Turner L., Scotton C., Negus R., Balkwill F. (1999). Hypoxia inhibits macrophage migration. Eur. J. Immunol..

[9] Grigorescu G.O., Preda M.B., Radu E., Rosca A.-M., Tutuianu R., Mitroi D.N., Simionescu M., Burlacu A. (2015). Combinatorial approach for improving the outcome of angiogenic therapy in ischemic tissues. Biomaterials.

[10] Lee E.J., Park H.-W., Jeon H.-J., Kim H.-S., Chang M.-S. (2013). Potentiated therapeutic angiogenesis by primed human mesenchymal stem cells in a mouse model of hindlimb ischemia. Regen. Med..

[11] Florczyk U., Jazwa A., Maleszewska M., Mendel M., Szade K., Kozakowska M., Grochot-Przeczek A., Viscardi M., Czauderna S., Bukowska-Strakova K. (2014). Nrf2 Regulates Angiogenesis: Effect on Endothelial Cells, Bone Marrow-Derived Proangiogenic Cells and Hind Limb Ischemia. Antioxid. Redox Signal..

[12] Ichihara S., Yamada Y., Liu F., Murohara T., Itoh K., Yamamoto M., Ichihara G. (2010). Ablation of the Transcription Factor Nrf2 Promotes Ischemia-Induced Neovascularization by Enhancing the Inflammatory Response. Arter. Thromb. Vasc. Biol..

[13] Graney P.L., Ben-Shaul S., Landau S., Bajpai A., Singh B., Eager J., Cohen A., Levenberg S., Spiller K.L. (2020). Macrophages of diverse phenotypes drive vascularization of engineered tissues. Sci. Adv..

[14] Peet C., Ivetic A., Bromage D., Shah A.M. (2020). Cardiac monocytes and macrophages after myocardial infarction. Cardiovasc. Res..

[15] Mantovani A., Biswas S.K., Galdiero M.R., Sica A., Locati M. (2013). Macrophage plasticity and polarization in tissue repair and remodelling. J. Pathol..

[16] Wynn T.A., Vannella K.M. (2016). Macrophages in Tissue Repair, Regeneration, and Fibrosis. Immunity.

[17] Dresske B., El Mokhtari N.E., Ungefroren H., Ruhnke M., Plate V., Janssen D., Siebert R., Reinecke A., Simon R., Fandrich F. (2006). Multipotent Cells of Monocytic Origin Improve Damaged Heart Function. Arab. Archaeol. Epigr..

[18] Berndt R., Hummitzsch L., Heß K., Albrecht M., Zitta K., Rusch R., Sarras B., Bayer A., Cremer J., Faendrich F. (2018). Allogeneic transplantation of programmable cells of monocytic origin (PCMO) improves angiogenesis and tissue recovery in critical limb ischemia (CLI): A translational approach. Stem Cell Res. Ther..

[19] Hummitzsch L., Zitta K., Rusch R., Cremer J., Steinfath M., Gross J., Fandrich F., Berndt R., Albrecht M. (2019). Characterization of the Angiogenic Potential of Human Regulatory Macrophages (Mreg) after Ischemia/Reperfusion Injury In Vitro. Stem Cells Int..

[20] Besnier M., Gasparino S., Vono R., Sangalli E., Facoetti A., Bollati V., Cantone L., Zaccagnini G., Maimone B., Fuschi P. (2018). miR-210 Enhances the Therapeutic Potential of Bone-Marrow-Derived Circulating Proangiogenic Cells in the Setting of Limb Ischemia. Mol. Ther..

[21] Spinetti G., Fortunato O., Caporali A., Shantikumar S., Marchetti M., Meloni M., Descamps B., Floris I., Sangalli E., Vono R. (2013). MicroRNA-15a and MicroRNA-16 Impair Human Circulating Proangiogenic Cell Functions and Are Increased in the Proangiogenic Cells and Serum of Patients With Critical Limb Ischemia. Circ. Res..

[22] Wang H.-W., Huang T.-S., Lo H.-H., Huang P.-H., Lin C.-C., Chang S.-J., Liao K.-H., Tsai C.-H., Chan C.-H., Tsai C.-F. (2014). Deficiency of the MicroRNA-31–MicroRNA-720 Pathway in the Plasma and Endothelial Progenitor Cells From Patients With Coronary Artery Disease. Arter. Thromb. Vasc. Biol..

[23] Gonsalves C.S., Li C., Mpollo M.-S.E.M., Pullarkat V., Malik P., Tahara S.M., Kalra V.K. (2015). Erythropoietin-mediated expression of placenta growth factor is regulated via activation of hypoxia-inducible factor-1α and post-transcriptionally by miR-214 in sickle cell disease. Biochem. J..

[24] Lee J.H., Yoon Y.M., Lee S.H. (2017). Hypoxic Preconditioning Promotes the Bioactivities of Mesenchymal Stem Cells via the HIF-1α-GRP78-Akt Axis. Int. J. Mol. Sci..

[25] Xia X., Chiu P.W.Y., Lam P.K., Chin W.C., Ng E.K.W., Lau J.Y.W. (2018). Secretome from hypoxia-conditioned adipose-derived mesenchymal stem cells promotes the healing of gastric mucosal injury in a rodent model. Biochim. Biophys. Acta BBA Mol. Basis Dis..

[26] Sierra-Parraga J.M., Merino A., Eijken M., Leuvenink H., Ploeg R., Møller B.K., Jespersen B., Baan C.C., Hoogduijn M.J. (2020). Reparative effect of mesenchymal stromal cells on endothelial cells after hypoxic and inflammatory injury. Stem Cell Res. Ther..

[27] Miceli V., Bulati M., Iannolo G., Zito G., Gallo A., Conaldi P.G. (2021). Therapeutic Properties of Mesen-chymal Stromal/Stem Cells: The Need of Cell Priming for Cell-Free Therapies in Regenerative Medicine. Int. J. Mol. Sci..

[28] Kale V., Vaidya A. (2020). Extracellular Vesicles Isolated from Mesenchymal Stromal Cells Primed with Hypoxia: Novel strategy in Regenerative Medicine. Curr. Stem Cell Res. Ther..

[29] Zhou P., Liu H., Liu X., Ling X., Xiao Z., Zhu P., Zheng S. (2021). Donor heart preservation with hypox-ic-conditioned medium-derived from bone marrow mesenchymal stem cells improves cardiac function in a heart transplantation model. Stem Cell Res. Ther..

[30] Zhang Z., Yao L., Yang J., Wang Z., Du G. (2018). PI3K/Akt and HIF 1 signaling pathway in hypoxia ischemia. Mol. Med. Rep..

[31] Sun J., Shen H., Shao L., Teng X., Chen Y., Liu X., Yang Z., Shen Z. (2020). HIF-1α overexpression in mesenchymal stem cell-derived exosomes mediates cardioprotection in myocardial infarction by enhanced angiogenesis. Stem Cell Res. Ther..

[32] Zhang Y., Hao Z., Wang P., Xia Y., Wu J., Xia D., Fang S., Xu S. (2019). Exosomes from human umbilical cord mesenchymal stem cells enhance fracture healing through HIF-1α-mediated promotion of angiogenesis in a rat model of stabilized fracture. Cell Prolif..

[33] Stather P.W., Wych J., Boyle J.R. (2019). A systematic review and meta-analysis of remote ischemic preconditioning for vascular surgery. J. Vasc. Surg..

[34] Lofano G., Mallett C.P., Bertholet S., O’Hagan D.T. (2020). Technological approaches to streamline vaccination schedules, progressing towards single-dose vaccines. NPJ Vaccines.

[35] Moshaverinia A., Chen C., Akiyama K., Xu X., Chee W.W.L., Schricker S.R., Shi S. (2013). Encapsulated dental-derived mesenchymal stem cells in an injectable and biodegradable scaffold for applications in bone tissue engineering. J. Biomed. Mater. Res. Part A.

[36] Jaluvka F., Ihnat P., Madaric J., Vrtkova A., Janosek J., Prochazka V. (2020). Current Status of Cell-Based Therapy in Patients with Critical Limb Ischemia. Int. J. Mol. Sci..

[37] Qadura M., Terenzi D.C., Verma S., Al-Omran M., Hess D.A. (2018). Concise review: Cell therapy for critical limb ischemia: An integrated review of preclinical and clinical studies. Stem Cells.

[38] Ludwinski F.E., Patel A.S., Damodaran G., Cho J., Furmston J., Xu Q., Jayasinghe S.N., Smith A., Modarai B. (2019). Encapsulation of macrophages enhances their retention and angiogenic potential. NPJ Regen. Med..

[39] Patel A.S., Smith A., Attia R.Q., Mattock K., Humphries J., Lyons O., Jayasinghe S.N. (2012). Encapsula-tion of angiogenic monocytes using bio-spraying technology. Integr. Biol..

[40] Choe G., Park J., Park H., Lee J.Y. (2018). Hydrogel Biomaterials for Stem Cell Microencapsulation. Polymers.

[41] Barre A., Naudot M., Colin F., Sevestre H., Collet L., Devauchelle B., Le Ricousse S. (2020). An algi-nate-based hydrogel with a high angiogenic capacity and a high osteogenic potential. BioRes. Open Access.

[42] Young S.A., Flynn L.E., Amsden B.G. (2018). Adipose-Derived Stem Cells in a Resilient In Situ Forming Hydrogel Modulate Macrophage Phenotype. Tissue Eng. Part A.

[43] Tong X., Yang F. (2018). Recent Progress in Developing Injectable Matrices for Enhancing Cell Delivery and Tissue Regeneration. Adv. Heal. Mater..

[44] Cheng N.-C., Lin W.-J., Ling T.-Y., Young T.-H. (2017). Sustained release of adipose-derived stem cells by thermosensitive chitosan/gelatin hydrogel for therapeutic angiogenesis. Acta Biomater..

[45] Fangradt M., Hahne M., Gaber T., Strehl C., Rauch R., Hoff P., Löhning M., Burmester G.-R., Buttgereit F. (2012). Human monocytes and macrophages differ in their mechanisms of adaptation to hypoxia. Arthritis Res. Ther..

[46] Gramley F., Lorenzen J., Pezzella F., Kettering K., Himmrich E., Plumhans C., Koellensperger E., Münzel T. (2009). Hypoxia and Myocardial Remodeling in Human Cardiac Allografts: A Time-course Study. J. Hear. Lung Transplant..

[47] Strehl C., Fangradt M., Fearon U., Gaber T., Buttgereit F., Veale D.J. (2014). Hypoxia: How does the mono-cyte-macrophage system respond to changes in oxygen availability?. J. Leukoc. Biol..

[48] Kumari R., Sunil D., Ningthoujam R.S. (2020). Hypoxia-responsive nanoparticle based drug delivery systems in cancer therapy: An up-to-date review. J. Control. Release.

[49] Zhou Y., Chen X., Cao J., Gao H. (2020). Overcoming the biological barriers in the tumor microenvironment for improving drug delivery and efficacy. J. Mater. Chem. B.

[50] Evans M.A., Shields C.W., Krishnan V., Wang L.L.W., Zhao Z., Ukidve A., Mitragotri S. (2020). Macrophage-Mediated Delivery of Hypoxia-Activated Prodrug Nanoparticles. Adv. Ther..

[51] Shields C.W., Evans M.A., Wang L.L.W., Baugh N., Iyer S., Wu D., Mitragotri S. (2020). Cellular back-packs for macrophage immunotherapy. Sci. Adv..

[52] Behfar A., Latere J.P., Bartunek J., Homsy C., Daro D., Crespo-Diaz R.J., Terzic A. (2013). Optimized de-livery system achieves enhanced endomyocardial stem cell retention. Circ. Cardiovasc. Interv..

[53] Chang H.-K., Kim P.-H., Kim N.W., Cho H.-M., Jeong M.J., Kim D.H., Joung Y.K., Lim K.S., Kim H.B., Lim H.C. (2018). Coronary stents with inducible VEGF/HGF-secreting UCB-MSCs reduced restenosis and increased re-endothelialization in a swine model. Exp. Mol. Med..

[54] Krishnagopal A., Reddy A., Sen D. (2017). Stent-mediated gene and drug delivery for cardiovascular disease and cancer: A brief insight. J. Gene Med..

[55] Panetta C.J., Miyauchi K., Berry D., Simari R.D., Holmes D.R., Schwartz R.S., Caplice N.M. (2002). A tissue-engineered stent for cell-based vascular gene transfer. Hum. Gene Ther..

[56] Kumar A.H., Martin K., Doyle B., Huang C.L., Pillai GK M., Ali M.T., Caplice N.M. (2014). Intravas-cular cell delivery device for therapeutic VEGF-induced angiogenesis in chronic vascular occlusion. Biomaterials.

[57] Osipova O., Saaya S., Karpenko A., Zakian S., Aboian E. (2019). Cell therapy of critical limb ischemia-problems and prospects. Vasa.

[58] Rafatian G., Davis D.R. (2018). Concise Review: Heart-Derived Cell Therapy 2.0: Paracrine Strategies to Increase Therapeutic Repair of Injured Myocardium. Stem Cells.

[59] Wang S.K., Green L.A., Motaganahalli R.L., Wilson M.G., Fajardo A., Murphy M.P. (2017). Rationale and design of the MarrowStim PAD Kit for the Treatment of Critical Limb Ischemia in Subjects with Severe Peripheral Arterial Disease (MOBILE) trial investigating autologous bone marrow cell therapy for critical limb ischemia. J. Vasc. Surg..

[60] Perin E.C., Murphy M., Cooke J.P., Moye L., Henry T.D., Bettencourt J., Gahremanpour A., Leeper N., Anderson R.D., Hiatt W.R. (2014). Rationale and Design for PACE: Patients with Intermittent Claudication Injected with ALDH Bright Cells. Am. Hear. J..

[61] Sprengers R., Moll F., Verhaar M. (2010). Stem Cell Therapy in PAD. Eur. J. Vasc. Endovasc. Surg..

[62] McCallum J.C., Lane J.S. (2014). Angiosome-directed revascularization for critical limb ischemia. Semin. Vasc. Surg.

[63] Iida O., Takahara M., Soga Y., Yamauchi Y., Hirano K., Tazaki J., Yamaoka T., Suematsu N., Suzuki K., Shintani Y. (2014). Impact of Angiosome-Oriented Revascularization on Clinical Outcomes in Critical Limb Ischemia Patients Without Concurrent Wound Infection and Diabetes. J. Endovasc. Ther..

[64] Naz I., Walters E., Akbari C.M., E Attinger C., Kim P.J. (2017). Noninvasive Vascular Assessment of Lower Extremity Wounds in Diabetics: Are We Able to Predict Perfusion Deficits?. Surg. Technol. Int..

[65] Krishnasamy K., Limbourg A., Kapanadze T., Gamrekelashvili J., Beger C., Häger C., Lozanovski V.J., Falk C.S., Napp L.C., Bauersachs J. (2017). Blood vessel control of macrophage maturation promotes arteriogenesis in ischemia. Nat. Commun..

[66] Stimpson A.L., Dilaver N., Bosanquet D.C., Ambler G.K., Twine C.P. (2019). Angiosome Specific Revascularisation: Does the Evidence Support It?. Eur. J. Vasc. Endovasc. Surg..

[67] Rigato M., Monami M., Fadini G.P. (2017). Autologous cell therapy for peripheral arterial disease: Systematic review and meta-analysis of randomized, nonrandomized, and noncontrolled studies. Circ. Res..

[68] Shin J.-Y., Yoon J.-K., Noh M.K., Bhang S.H., Kim B.-S., Noh M.M.K. (2016). Enhancing Therapeutic Efficacy and Reducing Cell Dosage in Stem Cell Transplantation Therapy for Ischemic Limb Diseases by Modifying the Cell Injection Site. Tissue Eng. Part A.

[69] Jin P., Wang E., Wang Y.-H., Huang W., Kuang W., Sun C., Hu S., Zhang H. (2012). Central zone of myocardial infarction: A neglected target area for heart cell therapy. J. Cell. Mol. Med..

[70] Jocius D., Vajauskas D., Skrebunas A., Gutauskas M., Tamosiunas A.E. (2019). Ischemic Muscle Necrosis of Lower Extremities in Peripheral Arterial Disease: The Impact of 99mTc-MDP Scintigraphy on Patient Management. Medicina.

[71] Liu M., Ma Z., Guo X., Zhu J., Su J. (2011). Technetium-99m-labelled HL91 and technetium-99m-labelled MIBI SPECT imaging for the detection of ischaemic viable myocardium: A preliminary study. Clin. Physiol. Funct. Imaging.

[72] Murdoch C.E., Shuler M., Haeussler D.J.F., Kikuchi R., Bearelly P., Han J., Watanabe Y., Fuster J.J., Walsh K., Ho Y.-S. (2014). Glutaredoxin-1 up-regulation induces soluble vascular endothelial growth factor receptor 1, attenuating post-ischemia limb revascularization. J. Biol. Chem..

[73] Matsui R., Watanabe Y., Murdoch C.E. (2017). Redox regulation of ischemic limb neovascularization—What we have learned from animal studies. Redox Biol..

[74] Watanabe Y., Murdoch C.E., Sano S., Ido Y., Bachschmid M.M., Cohen R.A., Matsui R. (2016). Glutathione adducts induced by ischemia and deletion of glutaredoxin-1 stabilize HIF-1α and improve limb revascularization. Proc. Natl. Acad. Sci. USA.

[75] Ho Y.-S., Xiong Y., Ho D.S., Gao J., Chua B.H.L., Pai H., Mieyal J.J. (2007). Targeted disruption of the glutaredoxin 1 gene does not sensitize adult mice to tissue injury induced by ischemia/reperfusion and hyperoxia. Free Radic. Biol. Med..

[76] Bachschmid M.M., Xu S., Maitland-Toolan K.A., Ho Y.-S., Cohen R.A., Matsui R. (2010). Attenuated cardiovascular hypertrophy and oxidant generation in response to angiotensin II infusion in glutaredoxin-1 knockout mice. Free Radic. Biol. Med..

[77] Wang Q., Wang K., Zhao X. (2021). Monocytes recruitment blocking synergizes with mesenchymal stem cell transplantation for treating myocardial infarction. Regen. Med..

[78] Hummitzsch L., Albrecht M., Zitta K., Hess K., Parczany K., Rusch R., Cremer J., Steinfath M., Haneya A., Faendrich F. (2020). Human monocytes subjected to ischaemia/reperfusion inhibit angiogenesis and wound healing in vitro. Cell Prolif..

[79] Forget A., Gianni-Barrera R., Uccelli A., Sarem M., Kohler E., Fogli B., Shastri V.P. (2019). Mechanically defined microenvironment promotes stabilization of microvasculature, which correlates with the enrichment of a Novel Piezo-1+ population of circulating CD11b+/CD115+ monocytes. Adv. Mater..

[80] Ozawa C.R., Banfi A., Glazer N.L., Thurston G., Springer M.L., Kraft P.E., McDonald D.M., Blau H.M. (2004). Microenvironmental VEGF concentration, not total dose, determines a threshold between normal and aberrant angiogenesis. J. Clin. Investig..

[81] Bergers G., Song S. (2005). The role of pericytes in blood-vessel formation and maintenance. Neuro Oncol..

[82] Au P., Tam J., Fukumura D., Jain R.K. (2008). Bone marrow–derived mesenchymal stem cells facilitate engineering of long-lasting functional vasculature. Blood.

[83] Murakami M. (2011). Signaling Required for Blood Vessel Maintenance: Molecular Basis and Pathological Manifestations. Int. J. Vasc. Med..

[84] Wietecha M.S., Cerny W.L., DiPietro L.A. (2012). Mechanisms of Vessel Regression: Toward an Understanding of the Resolution of Angiogenesis. Current Topics in Microbiology and Immunology.

[85] Koike N., Fukumura D., Gralla O., Au P., Schechner J.S., Jain R.K. (2004). Creation of long-lasting blood vessels. Nat. Cell Biol..

[86] Lemcke H., Gaebel R., Skorska A., Voronina N., Lux C.A., Petters J., Sasse S., Zarniko N., Steinhoff G., David R. (2017). Mechanisms of stem cell based cardiac repair-gap junctional signaling promotes the cardiac lineage specification of mesenchymal stem cells. Sci. Rep..

